# Refined protocols of tamoxifen injection for inducible DNA recombination in mouse astroglia

**DOI:** 10.1038/s41598-018-24085-9

**Published:** 2018-04-12

**Authors:** Hannah M. Jahn, Carmen V. Kasakow, Andreas Helfer, Julian Michely, Alexei Verkhratsky, Hans H. Maurer, Anja Scheller, Frank Kirchhoff

**Affiliations:** 1 Molecular Physiology, Center for Integrative Physiology and Molecular Medicine (CIPMM), University of Saarland, 66421 Homburg, Germany; 2 Faculty of Biology, Medicine and Health, The University of Manchester, Manchester M13 9PL, UK; 3 Department of Experimental and Clinical Toxicology, University of Saarland, 66421 Homburg, Germany; 4 Present Address: Cologne Excellence Cluster on Cellular Stress Responses in Aging-Associated Diseases (CECAD), University Hospital of Cologne, Joseph-Stelzmann-Str. 26, 50931 Cologne, Germany

## Abstract

Inducible DNA recombination of floxed alleles *in vivo* by liver metabolites of tamoxifen (TAM) is an important tool to study gene functions. Here, we describe protocols for optimal DNA recombination in astrocytes, based on the GLAST-Cre^ERT2^/loxP system. In addition, we demonstrate that quantification of genomic recombination allows to determine the proportion of cell types in various brain regions. We analyzed the presence and clearance of TAM and its metabolites (N-desmethyl-tamoxifen, 4-hydroxytamoxifen and endoxifen) in brain and serum of mice by liquid chromatographic-high resolution-tandem mass spectrometry (LC-HR-MS/MS) and assessed optimal injection protocols by quantitative RT-PCR of several floxed target genes (*p2ry1*, *gria1, gabbr1 and Rosa26-tdTomato locus*). Maximal recombination could be achieved in cortex and cerebellum by single daily injections for five and three consecutive days, respectively. Furthermore, quantifying the loss of floxed alleles predicted the percentage of GLAST-positive cells (astroglia) per brain region. We found that astrocytes contributed 20 to 30% of the total cell number in cortex, hippocampus, brainstem and optic nerve, while in the cerebellum Bergmann glia, velate astrocytes and white matter astrocytes accounted only for 8% of all cells.

## Introduction

**Figure 1:**
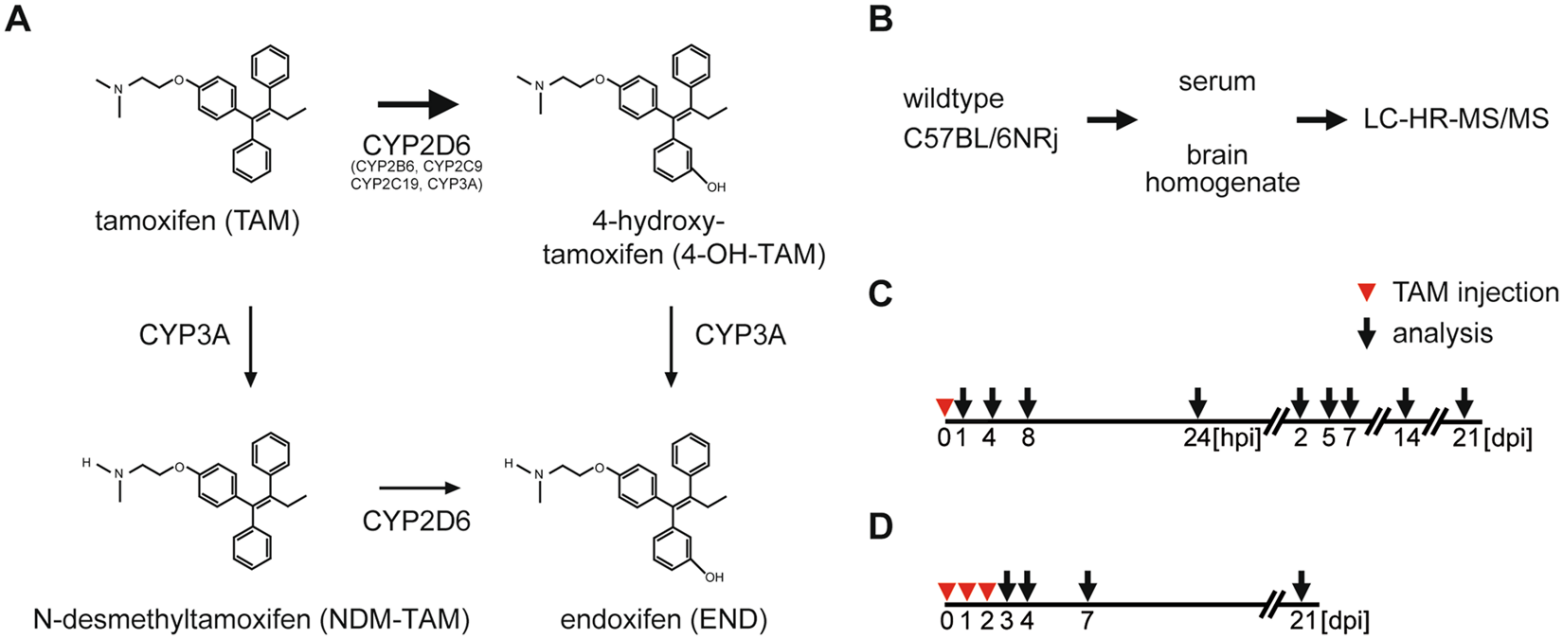
Tamoxifen metabolism and treatment protocols for LC-HR-MS/MS analysis. (**A**) Tamoxifen (TAM) is predominantly metabolized via two pathways. In the first, TAM is converted by CYP3A to N-desmethyl-tamoxifen (NDM-TAM), one of the major metabolites. This metabolite undergoes multiple oxidation steps including 4-hydroxylation to endoxifen (END) by CYP2D6. 4-Hydroxylation of TAM to 4-hydroxytamoxifen (4-OH-TAM) via multiple CYPs represents the second metabolic route, with significant importance for experimental biology in mice. A small proportion of endoxifen appears to result from CYP3A-catalyzed N-demethylation of 4-OH-TAM^10,11,49,50^. (**B**) Samples (serum/brain) of treated C57BL/6NRj wild type (wt) mice were collected and were analyzed by LC-HR-MS/MS. (**C**,**D**) Mice were injected on post-natal day 28 with TAM (100 mg/kg) once (**C**) or for three consecutive days (D, red triangles). Brain and blood samples were collected at different time points (indicated with black arrows). After processing samples were analyzed by LC-HR-MS/MS for TAM and its derivatives NDM-TAM, 4-OH-TAM and END.

Tamoxifen (TAM) has become an important reagent utilized for the analysis of gene functions in inducible conditional mouse mutants. TAM is oxidized in the liver by cytochrome P450 isoenzymes to N-desmethyl-tamoxifen (NDM-TAM), 4- hydroxytamoxifen (4-OH-TAM) and endoxifen (END)^1–3^ (Fig. 1A). TAM and its metabolites bind to estrogen receptors (ER) with different affinities. In humans, suppression of the estrogen-dependent cell proliferation by 4-OH-TAM is 30- to 100-fold higher compared to TAM^4–6^, although it represents less than 10% of all primary oxidation products of TAM^7^. END shares identical properties but is present in concentrations up to 10-fold higher than 4-OH-TAM^8,9^.

In mice, the pharmacokinetic profile of TAM appears significantly different, mainly due to the lower expression and activity levels of P450 isoenzymes^10,11^. Cell type-specific and temporally controlled recombination of floxed target genes is achieved by the Cre^ERT2^/loxP system. Cre^ERT2^ is a recombinant fusion protein of the Cre DNA recombinase and a mutant ligand-binding domain of the human estrogen receptor. Similarly to the endogenous estrogen receptor, Cre^ERT2^ is trapped in the cytosol by heat shock proteins. While this protein complex is impervious to natural ligands, it is highly sensitive to synthetic estrogen antagonists, such as 4-OH-TAM^12,13^ which liberate the Cre^ERT2^ for recombination of floxed alleles in the cell nucleus. The inducible Cre/loxP system is widely used for the generation of conditional gene-deficient mice or for the induction of cell type-specific reporter expression (www.jax.org, www.networkglia.eu/en/animal_models). To promote cost efficiency, scientists prefer TAM rather than the more expensive 4-OH-TAM for induction of gene recombination, taking advantage of its conversion to the bioactive metabolite 4-OH-TAM in the liver.

Three parameters mainly determine the specific and effective Cre-loxP recombination *in vivo*: (1) the dose and duration of TAM that determine the activity of the potent metabolites 4-OH-TAM and END, (2) the level of Cre^ERT2^ expression and (3) the chromatin structure and epigenetic status of the floxed alleles. Comparing the recombination efficiencies of published Cre driver lines is difficult, since the different labs use a wide range of application protocols (dose and preparation of TAM, e.g. oil suspensions vs. implanted pellets; time periods and routes of administration (subcutaneous vs. intraperitoneal vs. oral). Frequently, counting reporter positive cells is used to evaluate TAM induced recombination^14,15^. However, this approach strongly depends on the penetrance, i.e. the ubiquitous expression, of the reporter line^16,17^.

Focusing on astroglia, we combined the analysis of the pharmacokinetic profile of TAM and its metabolites by liquid chromatography-high resolution-tandem mass spectrometry (LC-HR-MS/MS) with the *in vivo* quantification of recombination events by quantitative real-time polymerase chain reaction (qRT-PCR) to reveal optimal protocols for cell type-specific DNA recombination. In addition, we demonstrate that qRT-PCR of cell-specific genomic recombination can be employed to quantify the proportion of cell types in a given brain tissue.

## Materials and Methods

### Ethics statements

Animal experiments were carried out at the University of Saarland according to European and German guidelines and approved by “Landesamt für Gesundheit und Verbraucherschutz” of Saarland state (license numbers: 72/2010, 65/2013 and 34/2016).

### Mice

Transgenic and wild type (wt) mice were maintained in the animal facilities of the Medical Faculty (University of Saarland). Mice received food *ad libitum*. Knockin GLAST-Cre^ERT2^ mice (Slc1a3^tm1(cre/ERT2)Mgoe^, MGI:3830051)^18^ were crossbred to mice with floxed target genes (P2Y1 receptor, *p2ry1*, Christian Gachet, INSERM U949, Strasbourg, France; GABA_B_ receptor subunit 1, Gabbr1^tm2Bet^, MGI:3512742^19^ and AMPA-type glutamate receptor subunit GluA1 *gria1*, Gria1^tm1Rsp^, MGI:3798452^20^). Recombination events were also monitored using Rosa26 reporter mice (Ai14, Gt(ROSA)26Sor^tm14(CAG-tdTomato)Hze^, MGI:3809524)^17^. For qRT-PCR and TAM pharmacokinetic analyses, 7–8 week-old transgenic or C57BL/6NRj wt mice were studied.

### Sample preparation

Anesthetized mice (ketamine/xylazine; 140 mg/kg and 10 mg/kg body weight) were perfused with ice-cold HBSS (Hank’s balanced salt solution w/o Ca^2+^ or Mg^2+^; Sigma, St. Louis, MO, USA) for 2–3 min. For the pharmacokinetic analysis of tamoxifen, blood was withdrawn from the right auricle (400–600 μl) before perfusion and centrifuged at 3000 rpm for 10 min to obtain the serum. After perfusion, brains were immediately cut sagittally, transferred into tubes with 500 μl 0.9% NaCl solution and homogenized (Precellys 24; peqlab, Erlangen, Germany). Samples were kept in light-protected 2 ml tubes at −80 °C till further analysis. For qRT-PCR, mice were perfused with HBSS. Brainstem (bs), cerebellum (cb), cortex (ctx), hippocampus (hc) and optic nerve (opt) were prepared, directly frozen and kept at −80 °C till further processing. To quantify reporter expression mice were perfused with artificial cerebral spinal fluid, followed by perfusion with 15 ml of 4% formaldehyde solution. Post-fixed vibratome sections (40 µm, Leica VT 1000 S, Leica Instruments, Nussloch, Germany) were prepared as described previously^21^. For immunohistochemical analysis, sagittal sections were stained against cell-specific marker proteins: rabbit anti-glial fibrillary acidic protein (GFAP) (1:500, DakoCytomation, Glostrup, Denmark), mouse anti-glutamine synthetase (GS) (1:500, BD, Franklin Lakes, NJ, USA) and rabbit anti-S100B (1:500, Abcam, Cambridge, UK). Nuclei were stained with TO-PRO-3 (1:10000, Invitrogen, Grand Island NY, USA). For overviews, sagittal sections were imaged with an epifluorescent, fully automated slide scanner (AxioScanZ.1; Zeiss Jena, Germany) equipped with an HBO lamp (HXP 120 V, LEJ, Jena, Germany), appropriate excitation and emission filter sets, a Plan-Apochromat 10×/0.45 objective for prefocussing and a Plan-Apochromat 20×/0.8 objective for fine focus image acquisition. Offline image stitching (8 µm stacks, variance projection, ZEN, Zeiss) was performed and processed with Fiji software^22^. Confocal laser scan images (LSM 710, Zeiss) were recorded with appropriate excitation lasers and emission filters using Achroplan 10×/0.25 and Plan-Neofluar 40×/1.34 Oil DIC objectives. z-Stacks of images were taken at 1 µm intervals, processed with Fiji and displayed as maximum intensity projections.

### LC-HR-MS/MS analysis

**Figure 2:**
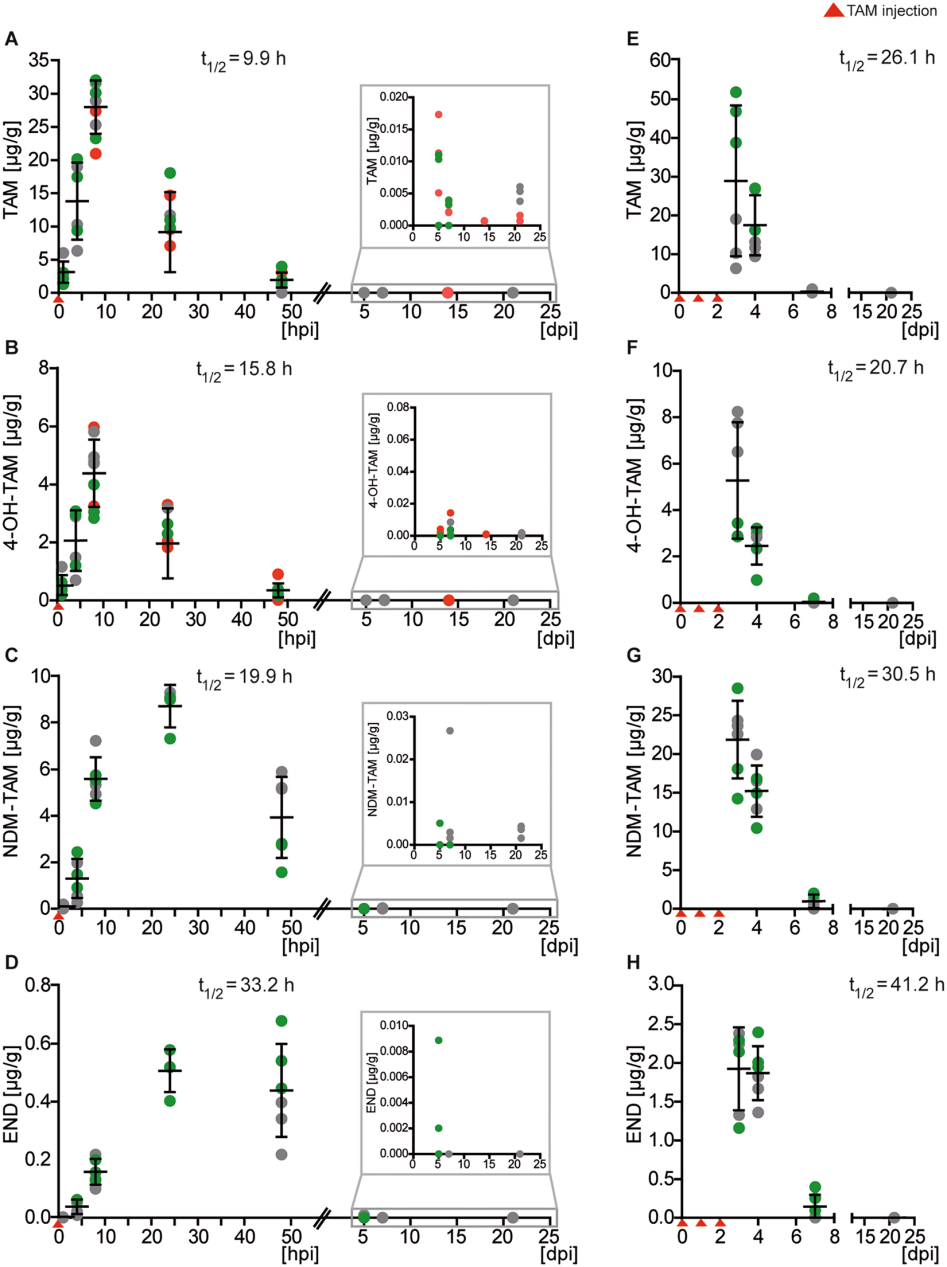
Fast accumulation and clearance of TAM and its metabolites in the brain. (**A**) TAM and (**B**) 4-OH-TAM peak at 8 hpi after one TAM injection with 6× more TAM than 4-OH-TAM and subsequent clearance to ineffective levels at 7 dpi. (**C**) NDM-TAM and (**D**) END concentrations peak after one additional TAM injection at 24 hpi with 17× more NDM-TAM than END, but are also cleared 7 dpi. Insets show the last three time points in higher resolution. Half-lives (t_1/2_) are indicated for each metabolite. Three consecutive TAM injections lead to a prolonged TAM (**E**), 4-OH-TAM (**F**), NDM-TAM (**G**) and END (**H**) enrichment in the brain with a delayed clearance compared to single TAM injections. The brain concentrations of all TAM derivatives are plotted against the time. Data are shown ± SEM with n = 3–9, depicted as single points and with colors (red, green and grey) indicating independent experiments.

Homogenized brain and serum samples were processed for LC-HR-MS/MS as established before^23^: 250 μl of samples were mixed with 25 μl risperidone 1 mg/L methanolic spike solution as internal standard and 750 μl zinc sulfate (ZnSO_4_) solution. Samples were vortexed for 2 min, stored for 5 min at −20 °C and centrifuged at 14,000 min^−1^. Supernatants (900 μl) were transferred to brown glass vials for further analysis using an Accela LC system controlled by Aria software version 1.6.3 and coupled to a high resolution Q-Exactive system equipped with a heated electrospray ionization (HESI)-II source and controlled by Xcalibur 2.2 SP1.48 software (all of ThermoFisher, Dreieich, Germany). Mass calibration was performed according to the manufacturer’s recommendations. Using a ThermoFisher Accucore PhenylHexyl column (100 mm × 2.1 mm I.D., 2.6 µm), guarded by an UHP filter cart (0.5 µm), liquid chromatography (LC) was performed at 35 °C (analytical column heater: HotDog 5090, Prolab, Reinach, Switzerland). The mobile phases consisted of 2 mM aqueous ammonium formate plus 0.1% formic acid (pH 3, eluent A) and 2 mM aqueous ammonium formate with acetonitrile:methanol (50:50, v/v; 1% water) plus 0.1% formic acid (eluent B). The flow rate was set to 0.5 mL/min for 10 min and 0.8 mL/min from 10–13.5 min. The gradient was programmed to: 0–1.0 min 99% A, 1–10 min to 1% A, 10–11.5 min hold 1% A, 11.5–13.5 min hold 99% A. The HESI-II source conditions were as follows: heater temperature, 320 °C; sheath gas, 60 arbitrary units (AU); auxiliary gas, 10 AU; spray voltage, 3.00 kV; capillary temperature, 32 °C; and S-lens RF level, 60.0. MS was performed in positive ionization mode using full scan (scan range, *m/z* 130–1,000; resolution, 35,000; microscans, 1; automatic gain control (AGC) target, 1e6; and maximum injection time (IT), 120 ms) and subsequent data dependent acquisition (resolution, 17,500; microscans, 1; AGC target, 2e5; maximum IT, 250 ms; loop count, 5; isolation window, *m/z* 1.0; high collision dissociation (HCD) cell stepped normalized collision energy (NCE), 17.5, 35, and 52.5%; spectrum data type, profile; intensity threshold, 4.0e3; dynamic exclusion, 8.0 s). Quantifications were performed with homogenized brain or serum sample calibrators spiked with defined concentrations of TAM, as well as its metabolites NDM-TAM, 4-OH-TAM and END (Fig. 2, Suppl. Information Fig. 2). The spiked concentrations were between 0.1 and 1,000 ng/mL with three separate calibration ranges (five calibrators each). Samples with concentrations above 1,000 ng/mL were diluted accordingly with blank sample.

### Tamoxifen-induced gene recombination

**Figure 3:**
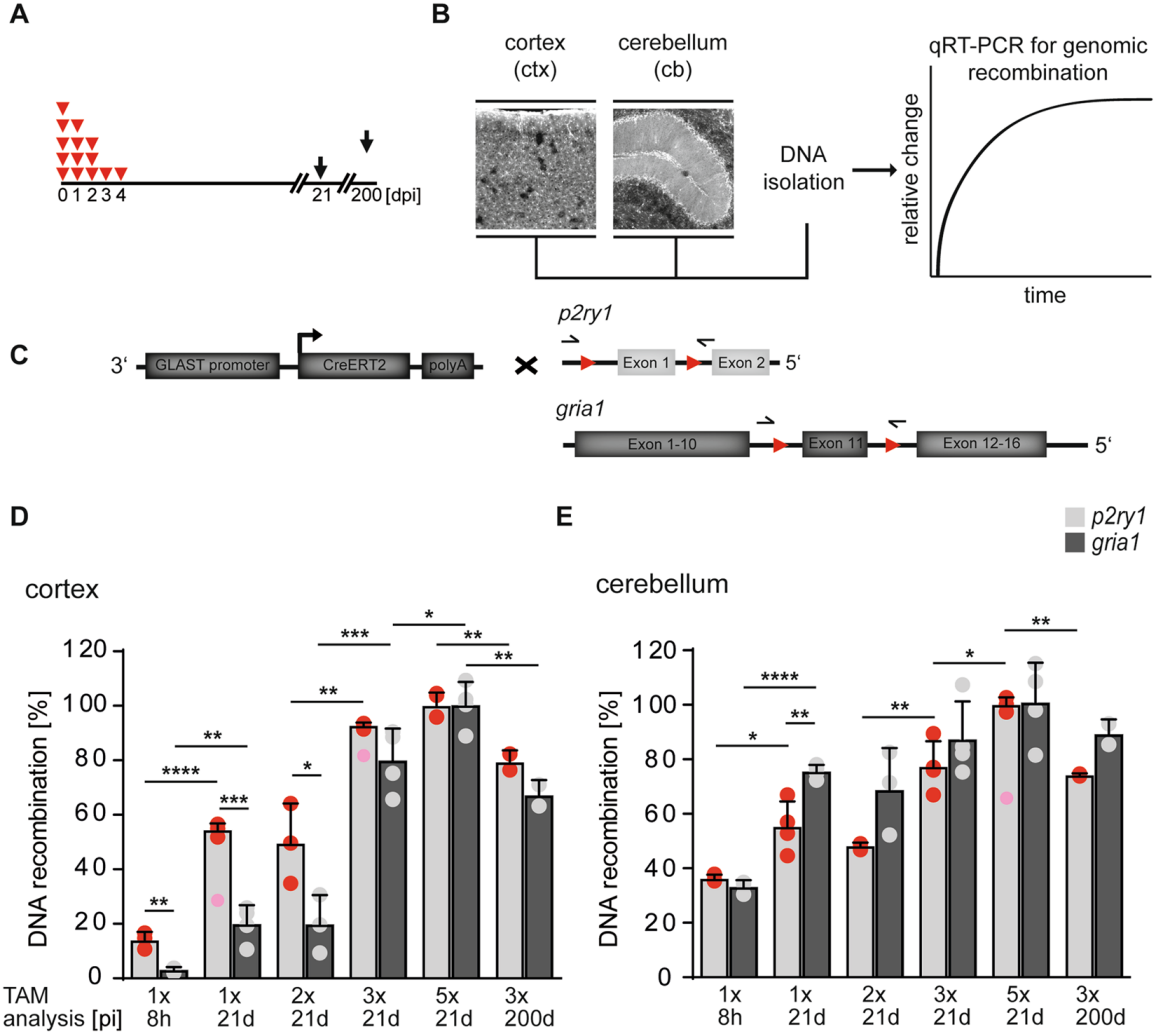
Three to five TAM injections give maximal DNA recombination depending on the brain region. (**A**) Different TAM injection protocols, varying from one single injection to five consecutive injections (red triangles), and analyzed at different time points (black arrows), were used to determine the most efficient protocol for recombination. (**B**,**C**) DNA from cortical and cerebellar tissue of GLAST-Cre^ERT2/+^ x P2Y1^fl/fl^ x GluA1^fl/fl^ mice was analyzed by quantitative RT-PCR (qRT-PCR). Primers (for *p2ry1* and *gria1* gene) span both loxP sites with amplification of the flanked sequence after recombination. (**D**,**E**) Differences in recombination efficiency between both analyzed alleles, but also both brain regions were detected with the lowest cortical and cerebellar recombination at 8 hpi. Per injection protocol three to four mice (exception 200 d, n** = **2) were analyzed (colored points) and ΔCT-values were normalized to the mean value of animals which received 5× TAM. The light red dots indicate significant outliers that were not considered for calculations. The error bars correlate to the SEM of the biological replicates (n** = **2–4, *p < 0.05, **p < 0.01, ***p < 0.001, ****p < 0.0001).

Transgenic mice were injected intraperitoneally with TAM in corn oil (100 mg/kg body weight, 10 mg/ml stock solution, Sigma, St. Louis, MO, USA) at the age of 4 weeks once per day for one, two, three or five consecutive days (Fig. 3A). In addition, mice were injected with TAM at different intervals (3× TAM with a pause of one day in between, Suppl. information Fig. 3A). Mice were analyzed 8 hours post injection (hpi), 48 hpi, 21 days post injection (dpi) or 200 dpi (Fig. 3A). For the pharmacokinetic analysis, wt mice received a single dose of TAM once or at three consecutive days (Fig. 1C,D). Samples were collected at 1 hpi, 4 hpi, 8 hpi, 24 hpi, 2 dpi, 5 dpi, 7 dpi, 14 dpi or 21 dpi. After three injections, tissue was collected 24 h after the last injection, corresponding to 3 d after the first injection. Similarly, when tissue samples were prepared at 18 d after the last injection, it corresponds to 21 d after the first injection (Fig. 1C,D). The biological half-lifes of TAM and its metabolites in blood or brain were determined after reaching maximum by non-linear regression (one-phase decay, GraphPad Prism 7, GraphPad Software Inc., La Jolla CA, USA). Over several years, we observed a mortality rate of 3.7% after three injections of TAM (n = 4.080, adult mice).

### DNA isolation

Genomic DNA was isolated using either the Invisorb Spin Tissue Mini Kit (Stratec Biomedical AG, Berlin, Germany) or the Qiagen, AllPrep DNA/RNA Mini Kit (Qiagen Inc., Valencia, CA, USA).

### Quantitative Real Time PCR (qRT-PCR)

Quantitative RT-PCR (qRT-PCR, CFX96 Real-Time PCR Detection System; BioRad, Hercules, CA, USA) was performed to determine (1) the loss of floxed (recombined) alleles (*loss*) in various brain regions and (2) relative increase of recombined alleles (*gain*) (ctx/cb) using EvaGreen for quantification (Axon, Kaiserslautern, Germany). *Loss* primers were located up- and downstream of the 5′ loxP site (*p2ry1*, *gabbr1*, *stop tdTomato)* to amplify non-recombined alleles. Values (ΔCT) of floxed recombined samples were normalized to those obtained from samples of control floxed non-recombined animals. *Gain* primers were designed to flank 3′ and 5′ loxP sites leading to PCR amplification after successful recombination. ΔCT values of floxed recombined mice were normalized to mean ΔCT values after five consecutive TAM injections. Reactions were carried out in triplicates with neuregulin 1 type III (*NrgIII*) and β-actin (*bact*) as endogenous gene controls. Data normalization and analysis (qbase+, Biogazelle, Gent, Belgium) was based on the ΔΔCT-method. Primers for qRT-PCR were as follows (in 5′ to 3′ direction): NrgIII-forward: GTG TGC GGA GAA GGA GAA AAC T; NrgIII-reverse: AGG CAC AGA GAG GAA TTC ATT TCT TA; β-actin-forward: CTG CTC TTT CCC AGA CGA GG, β-actin-reverse: AAG GCC ACT TAT CAC CAG CC; gabbr1-forward (loss): CAG TCG ACA AGC TTA GTG GAT CC; gabbr1-reverse (loss): TCC TCG ACT GCA GAA TTC CTG; p2ry1-forward (loss): CTT AGA TCG GTC GCA GCT CC; p2ry1-reverse (loss): GCG CTT TTG TCG CGT TAA TTA; tdTomato-forward (loss): ATC ATG TCT GGA TCC CCA TC, tdTomato-reverse (loss): CGT GGC CGT TCA TGG AGC CC; p2ry1-forward (gain): CTT AGA TCG GTC GCA GCT CC, p2ry1-reverse (gain): TGG CCA GTT TTC TTG GAG ACA; gria1-forward (gain): GTT CAG ACA GGG ACC CTC TCA; gria1-reverse (gain): GCC TGC CTG GGT AAA GTG ACT. For each primer pair, the efficiency was determined (Suppl. information Fig. 1).

### Statistical analysis

**Figure 4:**
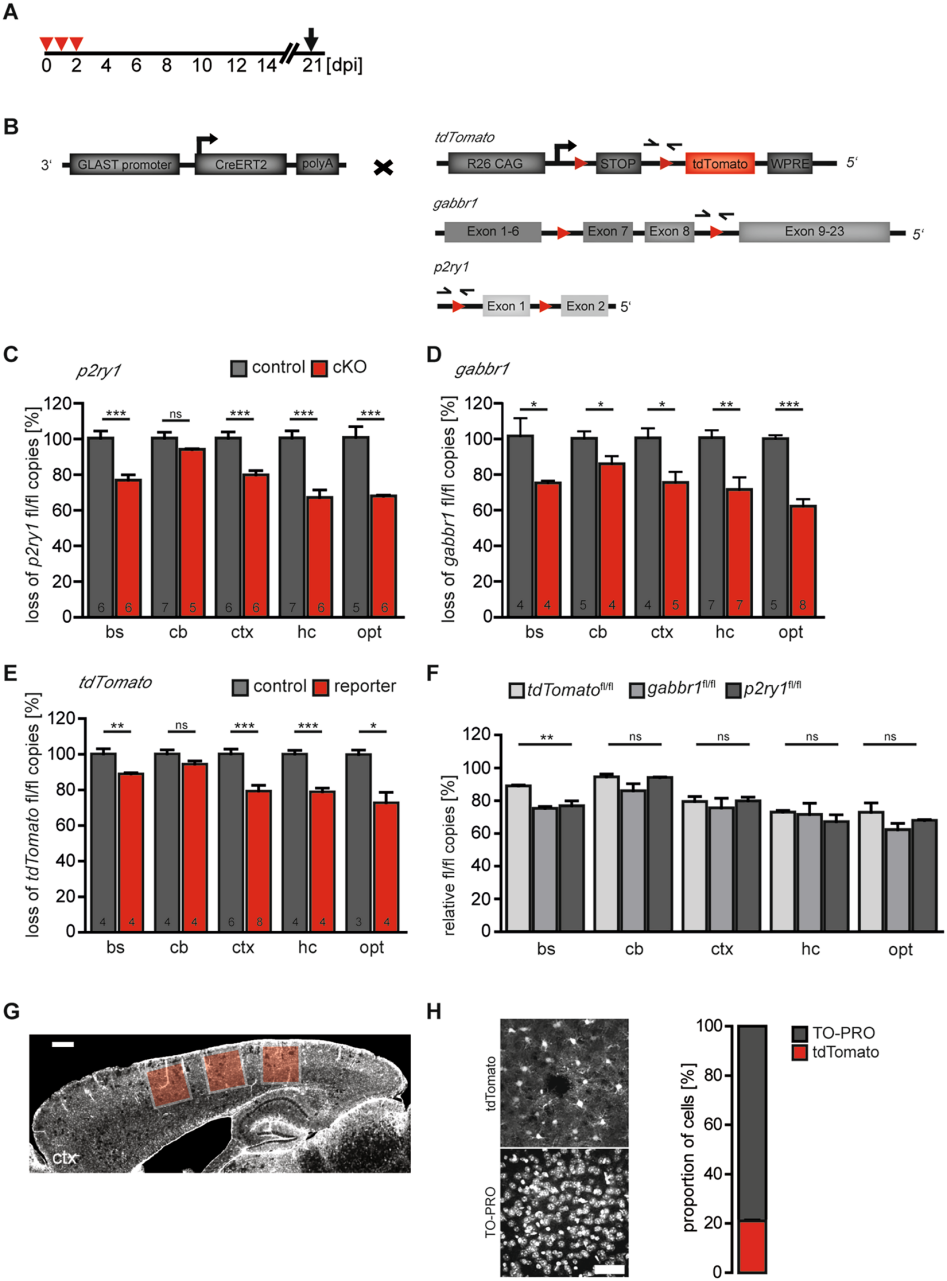
Comparison of recombination efficiencies at various gene loci. (**A**,**B**) Use of the mouse line GLAST-Cre^ERT2/+^ crossed to *stop*^fl/fl^ tdTomato, *gabbr1*^fl/fl^ and *p2ry1*^fl/fl^ mice for quantification of floxed, non-recombined alleles by qRT-PCR. Arrows indicate the position of primers after TAM injection for three consecutive days and analysis at 21 dpi. Reduction of floxed *p2ry1* alleles (**C**), floxed *gabbr1* (**D**) and floxed reporter (floxed stop cassette) (**E**) in bs, cb, ctx, hc and opt of transgenic animals compared to non-recombined alleles (100**%**) showed (**F**) no significant differences in all brain regions with the exception of the bs, where the reporter allele was recombined at a higher level than both floxed receptor genes. The error bars correlate to the SEM of the biological replicates (n = as indicated in bars (**C**–**E**), *p < 0.05, **p < 0.01, ***p < 0.001, unpaired t-test). (**G**) Micrograph of a representative cortical vibratome section prepared from a GLAST-Cre^ERT2/+^ x stop^fl/fl^ tdTomato mouse shows the extent of fluorescent recombined astrocytes. Colored squares represent the position of confocal images for stereological analysis. The scale bar corresponds to 500 µm. (**H**) Counting of TO-PRO-3+ and tdTomato + cells in cortices of GLAST-Cre^ERT2/+^ x stop^fl/fl^ tdTomato vibratome sections resulted in 21 (±3) % tdTomato positive cells. The scale bar correlates to 50 µm.

At least three animals were used per experiment and mouse line. All data were tested for normal distribution by the Shapiro-Wilk test. 3 datasets of the 24 experimental conditions of Fig. 3D and E as well as 1 out of 4 datasets from Suppl. Fig. 3 failed the test. Since they were of the same nature as the other 24, we regarded them as also being normally distributed and applied the Grubb’s test to identify outliers. These outliers were not included in the statistical calculations, but we depicted them in light red color to show the complete datasets. All data of Fig. 4 and Suppl. Fig. 7 were normally distributed.

Inter-group comparisons were performed by two-tailed Student *t* test using the GraphPad Prism 7 software (GraphPad Software Inc., La Jolla CA, USA). Data are represented as means ± SEM of natural replicates (mice) with *P < 0.05; **P < 0.01; ***P < 0.001; ****P < 0.0001. Significant outliers were not included into calculations. But, to demonstrate the full data-set, they were indicated by light red color in Fig. 3 and Suppl. information Fig. 3. tdTomato+ and TO-PRO-3+ cells were determined in three female and three male mice. A total of nine detailed images (from two parasagittal slices per animal; 40× objective, in posterior, mid and anterior regions of the cortex) were taken. No gender differences were observed.

### Data availability statement

The datasets generated during and/or analyzed during the current study are available from the corresponding author on reasonable request.

## Results

### Fast uptake and clearance of tamoxifen and its bioactive metabolite 4-OH-TAM into the brain

To analyze the contribution and time course of tamoxifen (TAM) and its metabolites N-desmethyl-tamoxifen (NDM-TAM), 4-hydroxytamoxifen (4-OH-TAM) and endoxifen (END) for cell type-specific recombination, we determined their pharmacokinetic profile by LC-HR-MS/MS. After a single intraperitoneal injection of TAM, metabolite concentrations were determined in serum and brain at 1, 4, 8, 24 h and 2, 5, 7, 14 and 21 d post injection (hpi or dpi) (Fig. 1B,C). In addition, the concentrations were determined after injection of TAM for three consecutive days (Fig. 1B,D).

TAM and 4-OH-TAM showed fast partitioning in the brain reaching maximal levels as early as 8 hpi. 4-OH-TAM entered the brain equally fast, although it required prior enzymatic hydroxylation of TAM in the liver by cytochrome P450 cyclooxygenases (Fig. 2). Corresponding TAM samples from the serum displayed profound variability, which was less pronounced for 4-OH-TAM (Suppl. information Fig. 2). We attributed this to variations of tissue and blood absorption after individual intraperitoneal injections. Both, TAM and 4-OH-TAM were efficiently cleared from brain and blood within 7 dpi (Suppl. information Table 1, Fig. 2A,B, Suppl. information Fig. 2A,B). In the brain, TAM was approximately six times enriched in comparison to 4-OH-TAM at 8 hpi as well as at 48 hpi (Fig. 2A,B). The other two metabolites, NDM-TAM and END, required three- to five-fold more time to reach their concentration peaks (at 24 hpi, Suppl. information Table 1, Fig. 2C,D, Suppl. information Fig. 2C,D). Nevertheless, both were cleared completely at 7 dpi (Fig. 2C,D, Suppl. information Fig. 2C,D). As expected from its lipophilic properties, TAM displayed the highest concentration in the brain followed by NDM-TAM, 4-OH-TAM and, at lowest, END (Fig. 2, Suppl. information Table 1).

Consecutive injections of TAM are used frequently to achieve higher recombination efficiencies. Therefore, we injected TAM for three consecutive days and determined the levels of TAM and its metabolites focusing at the time required for clearance (Fig. 2E–H). All four compounds showed the highest concentration 24 h after the last injection (3 dpi). While TAM and 4-OH-TAM were only slightly enriched, NDM-TAM and END were about 5-fold enriched in comparison to single injections (Fig. 2E–H, Suppl. information Table 1). Obviously, consecutive injections increase the levels of TAM metabolites in the brain.

For the brain we determined clearance rates (t_1/2_) of 9.9 h,15.8 h, 19.9 h and 33.2 h for TAM, 4-OH-TAM, NMD-TAM and END after a single injection, respectively. These rates increased after three injections to 26.1 h, 20.7 h, 30.5 h and 41.2 h. Although, concentration levels were highly variable in the serum, all four compounds were cleared with similar rate constants as in the brain (Suppl. information Fig. 2 and Table 1).

In summary, the time windows of optimal bioactivity of TAM and its metabolites are between 4 and 24 h post injection for single injections, and 4 h to 5 d for repeated injections over three consecutive days. All metabolites were effectively cleared to negligible levels at 7 dpi.

### Maximal recombination levels are reached by 3 or 5 days of TAM injections in cerebellum or cortex, respectively

To determine the *in vivo* efficacy of TAM injections to activate the Cre DNA recombinase, we studied the recombination of two floxed gene loci, *p2ry1* (P2Y1 receptor) and *gria1* (AMPA receptor subunit GluA1) in two different areas of the brain, cerebellum (cb) and cortex (ctx), using GLAST^CreERT2^ mice (high-affinity glutamate/aspartate transporter, GLAST, *slc1a3*; GLAST^CreERT2/+^ x gria1^fl/fl^ x p2ry1^fl/fl^; Fig. 3C). Mice received single injections of TAM (100 mg/kg body weight) on 1, 2, 3 and 5 consecutive days. Samples were analyzed at 8 hpi, 21 dpi and 200 dpi by qRT-PCR (*gain*), where successful recombination is indicated by an amplified PCR product (Fig. 3A,B). We also tested a two-day interval of three consecutive TAM injections (inspired by the rapid drop of 4-OH-TAM at 48 hpi) (Fig. 2B and Suppl. information Fig. 3).

After three TAM injections, both alleles (*p2ry1* and *gria1*) were already recombined at a percentage of 80 and more (n = 4, Fig. 3D,E, Suppl. information Table 2), in ctx as well as in cb. Almost 100% recombination was achieved after five consecutive injections. As expected from LC-HR-MS/MS data, increasing the time period between induction and analysis of recombination from 21 to 200 d did not change the recombination rate for both gene loci (n = 2, Suppl. information Table 2). Analyzing successful recombination already at 8 hpi after a single TAM injection revealed large differences in recombination within different brain regions, but also between different gene loci. In the ctx only a small percentage of floxed alleles was recombined (n = 3), while the recombination rate in the cb (n = 3) was higher. As expected from the longer bioavailability of its metabolites, higher degrees of recombination were observed at 21 dpi in comparison to 8 hpi (n = 3) after a single TAM injection (Fig. 3D,E, Suppl. information Table 2). Similarly, three TAM injections were more efficient than only two (n = 3) at 21 dpi (Fig. 3D,E, Suppl. information Table 2).

Since we observed fast clearance within less than 48 h, an interval protocol with one-day pause between injections (i.e. injections at every 2^nd^ day) was expected to be less efficient than a daily-injection protocol (Fig. 2, Suppl. information Fig. 3). Indeed, the interval protocol revealed less recombination in the ctx, (reduction of 32% for *p2ry1* and 61% for *gria1*) in comparison to the five-consecutive-day injection protocol (n = 4, Suppl. information Fig. 3B). In cb, the interval protocol proved to be less efficient than the 5-consecutive-day protocol (by 20%). But, in general, cerebellar recombination was higher than cortical recombination (Suppl. information Fig. 3C).

At early time points after TAM injections, (8 hpi) large differences in recombination efficiencies between different alleles and brain regions were detected. In the cortex, recombination of the *p2ry1* alleles was more than four times larger than *gria1* alleles. In contrast, in the cerebellum no difference between the two alleles was detected (Fig. 3D,E, Suppl. information Table 2). Simultaneously, the recombination rate of the cerebellum was much higher than in the cortex. At 21 dpi, a single TAM injection induced more than twice as many recombined *p2ry1* than *gria1* alleles in the ctx. This difference disappeared, when three consecutive injections were applied (Fig. 3D,E). In general, both target genes were recombined faster in the cb than in the ctx after one or two injections, while comparable recombination was observed after three and five injections. Maximal levels of recombination require single TAM injections for 3 to 5 d depending on brain region and floxed allele.

**Figure 5:**
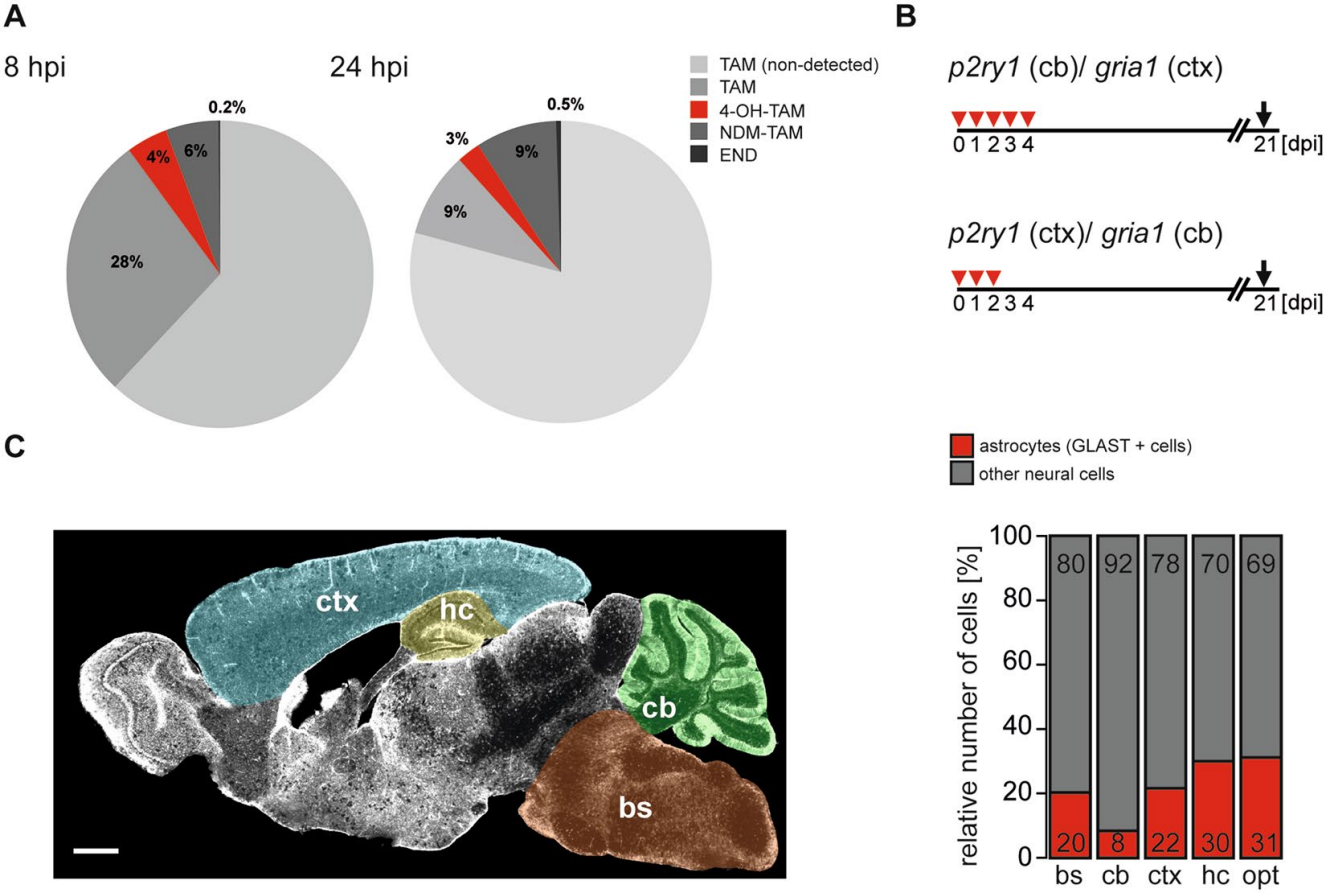
Inducible DNA recombination in mouse astroglia: Summary. (**A**) Concentrations of TAM and its derivatives after single TAM injections revealed that about 28**%** of the originally injected TAM could be detected in the brain while only 4**%** 4-OH-TAM, 6**%** NDM-TAM and negligible amount of END could be found at 8 hpi. While TAM (9**%**) and 4-OH-TAM (3**%**) decreased 24 hpi, NDM-TAM (9**%**) and END (3 × higher) increased their concentrations in the brain. (**B**) The optimized injection protocol for GLAST-Cre^ERT2^ mice for cortical and cerebellar recombination requires TAM injections for three to five consecutive days depending on brain region and floxed allele. (**C**) In a brain of a young adult mouse, astrocytes account for 20% (±2, n = 14) in the bs, 8% (±2, n = 13) in the cb, 22% (±2, n = 19) in the ctx, 30% (±3, n = 16) in the hc and 31% (±2, n = 17) in the optic nerve (setting GLAST-positive cells as astrocytes).

To functionally evaluate recombination efficiencies, we induced expression of the fluorescent reporter protein tdTomato in GLAST-Cre^ERT2^ x R26-tdTomato (stop^fl/fl^ tdTomato) mice (Suppl. information Figs 4, 5 and 6) and co-stained with astrocytic markers (GS, S100B, GFAP). After three consecutive injections, increasing numbers of recombined astrocytes could be identified throughout the brain (Suppl. information Figs 5 and 6). At 3.5 and 5 months post injection, a stabilized percentage of recombined cells was observed. The recombination specificity was the same in older mice (Supp. information Fig. 6). These data confirmed that three to five injections are sufficient to recombine the maximum number of cells in GLAST-Cre^ERT2^ mice of any age (Suppl. information Fig. 4).

### GLAST-Cre^ERT2^ mediated DNA recombination can be used to quantify astroglial cell numbers

If Cre^ERT2^ is expressed in a cell type-specific manner, the proportion of this cell type in a given region can be determined by qRT-PCR across a single loxP site of the non-recombined locus, since reduction of amplimer levels indicates successful gene deletion (Fig. 4, loss). Using the GLAST-Cre^ERT2^ mouse line, we compared recombination of three different floxed alleles (*stop*^*fl/fl*^ *tdTomato*; *p2ry1*^fl/fl^ and *gabbr1*^fl/fl^) in different brain regions (bs, cb, ctx, hc, opt).

Analyzing the floxed *p2ry1* alleles revealed recombination rates between 6% and 33% in the brainstem (bs), cb, ctx, hippocampus (hc) and optic nerve (opt) with similar  numbers for *gabbr1* and *tdTomato* alleles (Fig. 4C–E). No significant differences were found for most brain regions (cb (8 ± 2%), ctx (22 ± 2%), hc (30 ± 3%) and opt (31 ± 2%) (Fig. 4F)). Based on these data, the percentage of GLAST-Cre^ERT2^-positive cells (a rough estimate of astrocytes) varied from 6 to 30%, dependent on the brain region.

To confirm the qRT-PCR analysis, we also counted the number of recombined tdTomato-positive cells in the cortex (Fig. 4G,H). We found 21 ± 5% of all cells (TO-PRO labelled nuclei) were tdTomato-positive (Fig. 4H), a value well in-line with the observed cortical recombination (20 ± 2%). In addition, no significant differences of tdTomato-positive cells could be detected in the cortices of males and females (21 ± 6%, 20 ± 4%, respectively, Suppl. information Fig. 7). Our results show that qRT-PCR data of recombined loxP sites obtained from tissue homogenates correlates to counting of individual recombined cells. Therefore, the recombination rate reflects well the percentage of GLAST-positive cells (=astrocytes) in the respective brain regions.

## Discussion

The inducible Cre^ERT2^/loxP system is a highly effective genetic tool. By quantifying the bioavailability of tamoxifen (TAM) and its metabolites and by assessing genomic recombination using the GLAST-Cre^ERT2^ knockin mouse line, we provide a rationale for optimal protocols of recombination (Fig. 5). In addition, we show that qRT-PCR of the Cre^ERT2^/loxP system can also be used to determine the proportion of defined cell types in various tissue regions (Fig. 5).

Our main goal was to establish a protocol with the best recombination efficiency using the lowest TAM concentration to minimize side effects. For that purpose, we analyzed the concentration of TAM and its metabolites in the brain tissue and serum of wt mice. TAM and 4-OH-TAM showed highest concentrations already very early at 8 hpi, while NMD-TAM and END peaked at 24 hpi. We found that at  8 hpi,a single injection of TAM generated high levels of the effective 4-OH-TAM (4.4 µg/g brain weight), while others^24^ observed only 2.1 µg/g at 6 hpi and required an additional injection. Consecutive injections of low doses of TAM let all four substances accumulate, but they were efficiently excreted within 7 dpi, similar to a recent study, where different doses of TAM (up to 400 mg/kg body weight within 24 h) were compared^24^. Within the first 40 h, TAM and its metabolites were reduced by half after a single dose of TAM with TAM (9.9 h), 4-OH-TAM (15.8 h) and NMD-TAM (19.9 h) being cleared faster than END (33.2 h). Although TAM itself can be toxic, we observed a rather low mortality rate of 3.7% with a TAM concentration of 100 mg/kg, tested in many adolescent and adult mice (older than 21 days, n = 4,080). All results were obtained after intraperitoneal TAM injection which we prefer over oral gavage, due to the faster application and more precise dosage. The handling itself is also less stressful to the mice^24–26^. Others have also demonstrated a more efficient Cre activity in GLAST-CreERT2 knockin mice after i.p. injection of tamoxifen when compared to oral gavage^18^.

The minimal effective concentration of 4-OH-TAM has been determined at 38 ± 22 ng/g^24^. This value is slightly lower than the concentration we detected in the brain still at seven days when TAM was injected for three consecutive days (58.4 ng/g, 7 dpi). Long presence of 4-OH-TAM is important for genetic experiments in which extended gene excision is required. For fate mapping experiments, often short time windows for reporter gene activation are preferred. Our analysis demonstrates that such activation windows are shorter than five days after a single injection. At 5 dpi, only ineffective concentrations of 4-OH-TAM remain in the brain (1.3 ng/g).

The metabolites END and 4-OH-TAM have a 100 times stronger affinity to estrogen receptors in comparison to TAM^6,27,28^; and both are almost comparable in their induction capability of CreERT2-mediated gene recombination^29,30^. Since we found higher levels of END (up to two-fold) in comparison to 4-OH-TAM after 5 to 7 dpi, at this time some recombination due to slow accumulation of END cannot be excluded.

Three to five days of consecutive injections of TAM induced maximal recombination, while fewer injections were less effective. Similarly ineffective was a protocol with a pause between injections (three injections with 48 h in between) since TAM and 4-OH-TAM were cleared quickly, as seen by LC-HR-MS/MS. Differences of gene recombination at different floxed alleles or brain regions disappear after 21 days, when at least three injections are given. Because of its solubility properties, TAM has to be dissolved in oil. Due to variable partitioning, we observe strong fluctuations of TAM in serum of different animals. However, in the brain, the levels of TAM and its metabolites vary significantly less, probably due to the regulatory function of the blood-brain barrier.

Our data show that the three-to-five-injection standard protocol gives maximal recombination levels with a low mortality rate of less than 4%.

Astrocytes are often considered the most abundant cell type in the brain, but precise numbers are missing. Approaches used in recent work cannot distinguish different neuronal or glial cell types. Only ratios of neuronal (NeuN-positive) and non-neuronal (NeuN-negative = glial) cells were estimated in different brain regions of various species^31–35^ with about 50% of cells being glia in the mouse cerebral cortex. Inspired by our DNA recombination study, we provide an alternative approach that allows quantifying the proportion of individual glial cell populations. By continuing to use the GLAST-CreERT2 mice, we estimated the proportion of astrocytes in different regions of the murine brain. By setting the percentage of GLAST-CreERT2 mediated loss of floxed alleles as a measurement for the proportion of astrocytes (GLAST + cells) in a given brain tissue, we found 20% (brainstem), 8% (cerebellum), 22% (cortex), 30% (hippocampus) and 31% (optic nerve) of all cells account for astrocytes. In support of these results, direct cell counting of tdTomato + cells in the cortex revealed that 21% of all cells are astrocytes. In our experiments, we confirmed the high cell specificity of the GLAST-CreERT2 mouse line as extensively described^18^. Therefore, we interpreted GLAST + cells as astrocytes and neglected the different patterns of GLAST expression in astrocyte subpopulations or expression in neurogenic radial glia^36^. Regional differences of GLAST expression, for example in radial glial cells in the hippocampus (leading to recombined newly born granule cells), are not taken into account in our data sets^37,38^. Similar to our data, counting of S100B + cells in rat cortex revealed 18.5% astrocytes^39^. Our method relies on the fact that each cell contains the same genomic content including loxP sites. This approach can similarly be used for other glial cell types such as oligodendrocytes or microglia taking advantage of respective Cre DNA recombinase driver lines, e.g. by employing PLP-CreERT2 or CX3CR1-CreERT2^40,41^ mouse lines (see also http://www.networkglia.eu/en/animal_models). We tested our approach at three different floxed alleles: *p2ry1, gabbr1, tdTomato*. Although, largely, the three loci displayed very similar recombination efficiencies, we observed differences particularly at the onset of genomic recombination (*p2ry1, gria1*). Such differences disappeared when plateau phases of maximal recombination were reached.

One caveat is that the various gene loci may have different recombination efficiencies. Recombination kinetics for the AMPA receptor locus *gria1* was determined for the cerebellum on a precise time scale^42^. After 11 h about 50% of the recombination events occurred, i.e. three hours after the determined peak of TAM and 4-OH-TAM. In line with these data, we found that 33% recombined alleles for *gria1* and 36% for the *p2ry1* locus at 8 hpi. In contrast to the cerebellum, however, recombination in the cortex was lower with only 3% and 14% for *gria1* and *p2ry1*, respectively. Since effective recombination does not only depend on the level of Cre^ERT2^ expression, but also on chromatin structure, we suggest that the differences in recombination kinetics between (1) *gria1* and *p2ry1* and (2) the different brain regions (ctx vs. cb) are caused by different chromatin structures, with the *gria1* locus being less accessible than the *p2ry1* locus. Local chromatin structure and consequently, the potential for gene expression, are regulated by a number of post-translational, covalent modifications of histone-amino terminals, like methylation or acetylation^43–45^. Conceptually, within euchromatin structures the degree of condensation or DNA accessibility varies depending on gene activity^46^. Different DNA modifications between *p2ry1* and *gria1*, but also between ctx and cb could lead to different recombination efficiencies, as it has been shown for the Huntington’s disease homolog allele (*hdh*), which recombined only in the brain and in testis; both tissues with highest levels of hdh mRNA in mice^47^. In contrast, usage of the same Cre-expressing line caused recombination in every tissue when other floxed alleles were used^47^. Hence, for each new allele-of-interest a thorough DNA recombination analysis should be performed to rensure maximal recombination.

In this study, we focused on young adult mice. It is quite conceivable to assume that recombination varies with aging. And indeed, it has been proposed that tamoxifen induces recombination more efficiently in younger mice^48^. However, when we studied reporter expression in adult Rosa26-reporter mice using different Cre^ERT2^ expressing mouse lines, we could not detect changes in age-dependent recombination efficiency or specificity. In contrast, we are convinced that epigenetic changes affect the chromatin structure at distinct loci and thereby determine recombination. The activity level of a given locus, more than age, gender or strain background itself, determine recombination, as discussed above. Therefore, our work is designed to offer a blueprint for urgently required future experiments that address the interdependence of physiological requirements at different ages, different animal activity levels, different gender and the activity of chromatin modifying enzymes.

## Electronic supplementary material

Supplementary Information
