# Supplementary material for: Refined protocols of tamoxifen injection for inducible DNA recombination in mouse astroglia
Source: Sci Rep. 2018 Apr 12;8:5913. doi: 10.1038/s41598-018-24085-9 (PMC5897555; doi:10.1038/s41598-018-24085-9)
Supplement: Supplementary file 1 — Supplementary Information [file 41598_2018_24085_MOESM1_ESM.pdf]

Refined protocols of tamoxifen injection for inducible DNA recombination in mouse astroglia

Hannah M. Jahn[1,2,3,#], Carmen V. Kasakow[1,#], Andreas Helfer[4], Julian Michely[4], Alexei Verkhratsky[2], Hans H. Maurer[4], Anja Scheller[1] and Frank Kirchhoff[1,*]

[1] Molecular Physiology, Center for Integrative Physiology and Molecular Medicine (CIPMM), University of Saarland, Homburg, Germany

[2] Faculty of Biology, Medicine and Health, The University of Manchester, Manchester, UK

[3] Current address: Cologne Excellence Cluster on Cellular Stress Responses in Aging-Associated Diseases (CECAD), University Hospital of Cologne, Joseph-Stelzmann-Str. 26, Cologne

[4] Department of Experimental and Clinical Toxicology, University of Saarland, 66421 Homburg, Germany

[#] equal contribution

For correspondence:

* Frank Kirchhoff: Molecular Physiology, Center for Integrative Physiology and Molecular Medicine, University of Saarland, 66421 Homburg, Germany

Email: frank.kirchhoff@uks.eu

Phone: +49 6841 1616440

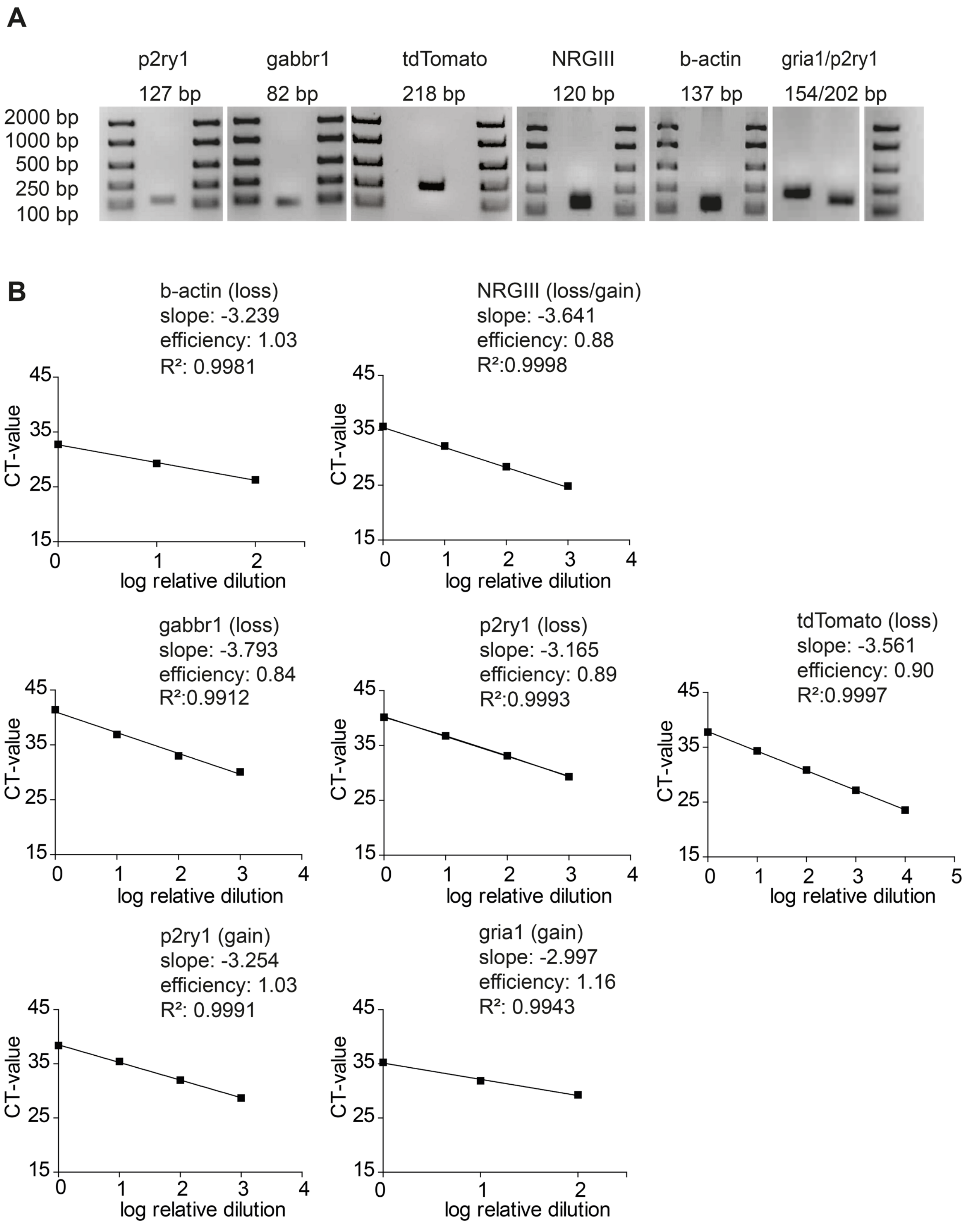

Supplementary information Figure 1: Primer efficiencies and test of primer specificity. (A) Primers showed single bands at the correct size after gel electrophoresis after qRT-PCRs. (B) Efficiencies were determined for β-actin (loss), NRGIII (loss/gain), tdTomato (loss), gabbr1 (loss), p2ry1 (loss), p2ry1 (gain) and gria1 (gain). PCRs were run on undiluted, 1:10, 1:100 and 1:1000 diluted samples. Threshold cycles of the dilution were determined and plotted inversely versus the logarithm of the dilution. Data were fitted with a linear regression. The slope was used to determine exponential amplification 10(-1/slope), efficiency [10(-1/slope)]–1 of the PCRs and the R2 value as indicated.

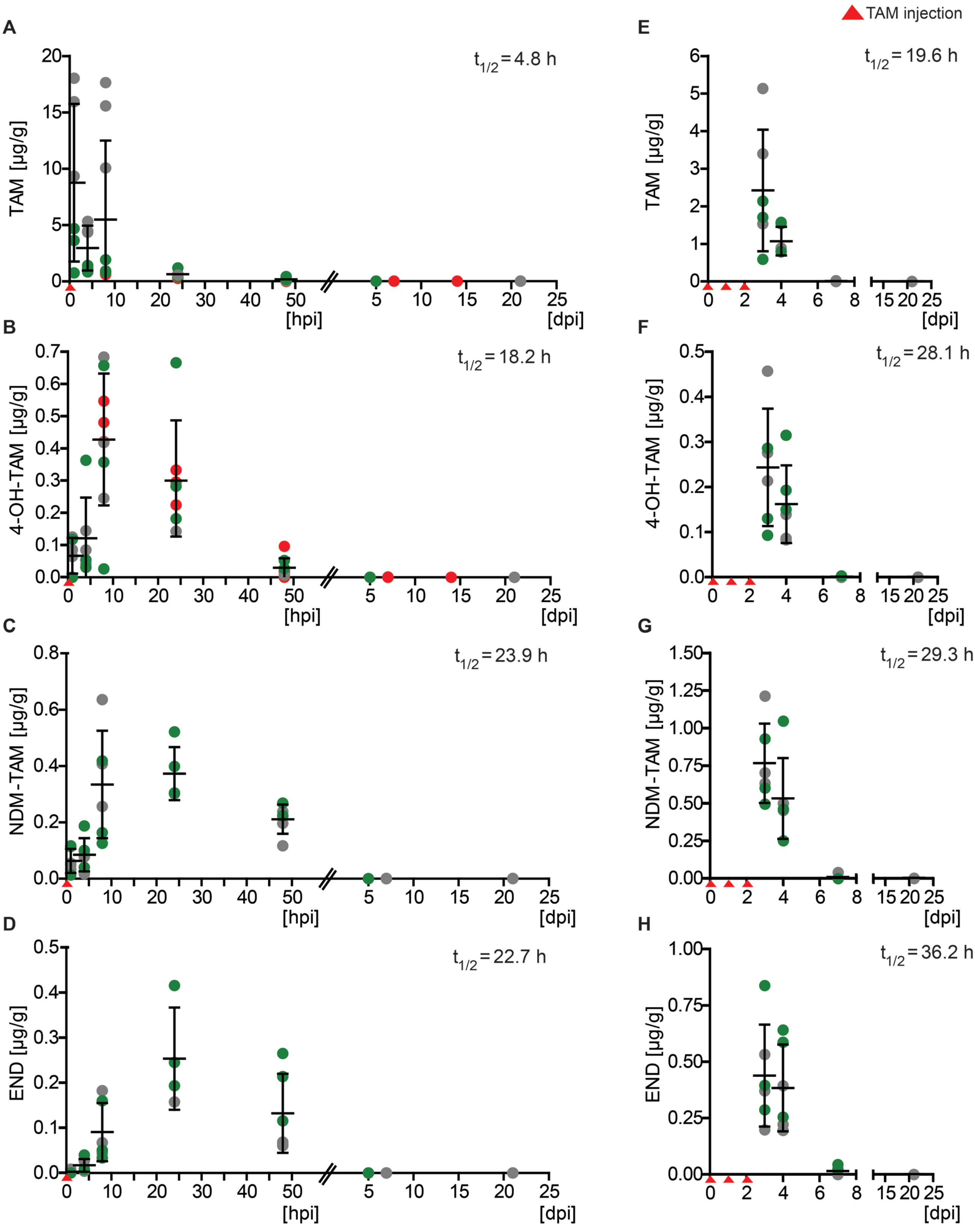

Supplementary information Figure 2: Serum concentrations of TAM and its metabolites. (A) Levels of TAM were highly variable after the first 8 hpi in serum after a single TAM injection, while after 24 hpi more uniform levels could be detected. (B) 4-OH-TAM peaked 8 hpi and showed subsequently lower levels at 5 dpi. (C) NDM-TAM and (D) END concentrations peak after one TAM injection at 24 hpi with only slightly more NDM-TAM than END. Both are also cleared equally well after 7 dpi. Three consecutive TAM injections lead to a higher concentration of TAM (E), 4-OH-TAM (F), NDM-TAM (G) and END (H) up to 48 hpi in the serum with longer half-lives compared to single TAM injections. The serum concentrations of all TAM derivatives are plotted against time. Data are shown ± SEM with n = 3-9, depicted as single closed circles and with colors (red, green and grey) indicating independent experiments.

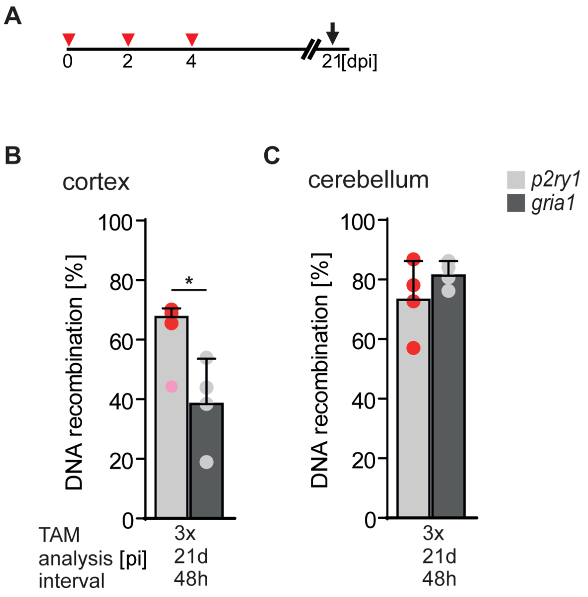

Supplementary information Figure 3: Interval injections of TAM with a day pause in between leads to insufficient recombination levels. (A) TAM injections for three days with a pause of 48 h in between injections (red triangles) leads to (B) lower recombination of gria1 compared to p2ry1 after 48 h in cortical astrocytes. (C) Cerebellar recombination shows no significant difference between gria1 and p2ry1. Four animals were analyzed (colored dots) and ΔCT-values were normalized to the mean value of animals which received 5x TAM (data are from Fig. 3D, E of the main manuscript). The light red dot is a statistical outlier according to Grubb's test and was not included into statistical calculations. It is depicted to show all experimental data. The error bars correlate to the SEM of the biological replicates (n = 4, *p<0.05, **p< 0.01, ***p<0.001, unpaired t-test).

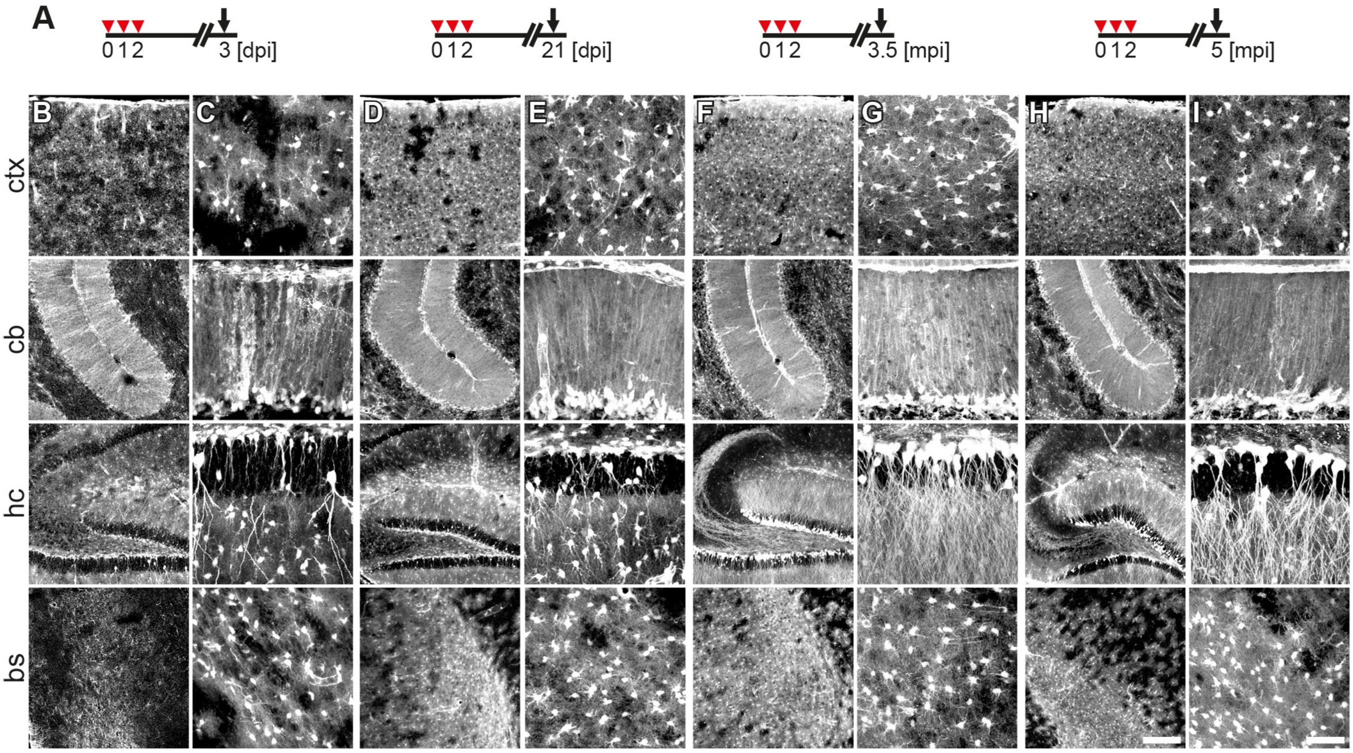

Supplementary information Figure 4: Wide reporter expression can already be detected at 3 dpi. It remains stable for at least 5 months after TAM injections. (A) For the determination of efficient recombination, TAM was injected for three consecutive days (indicated by red triangles) and different brain regions were analyzed at different time points (black arrows, 3 dpi, 21 dpi, 3.5 mpi and 5 mpi). Fluorescent protein expression after recombination in cortical grey matter astrocytes, cerebellar Bergman glia, hippocampal astrocytes and granule cells in the dentate gyrus (newly born neurons) and astrocytes in the brain stem at 3 dpi (B, C), with an increase at 21 dpi (D, E), a stable expression after 3.5 mpi (F, G) and a comparable expression 5 mpi (H, I). Maximum recombination was achieved already at 21 dpi. Scale bars indicate 200 and 50 µm.

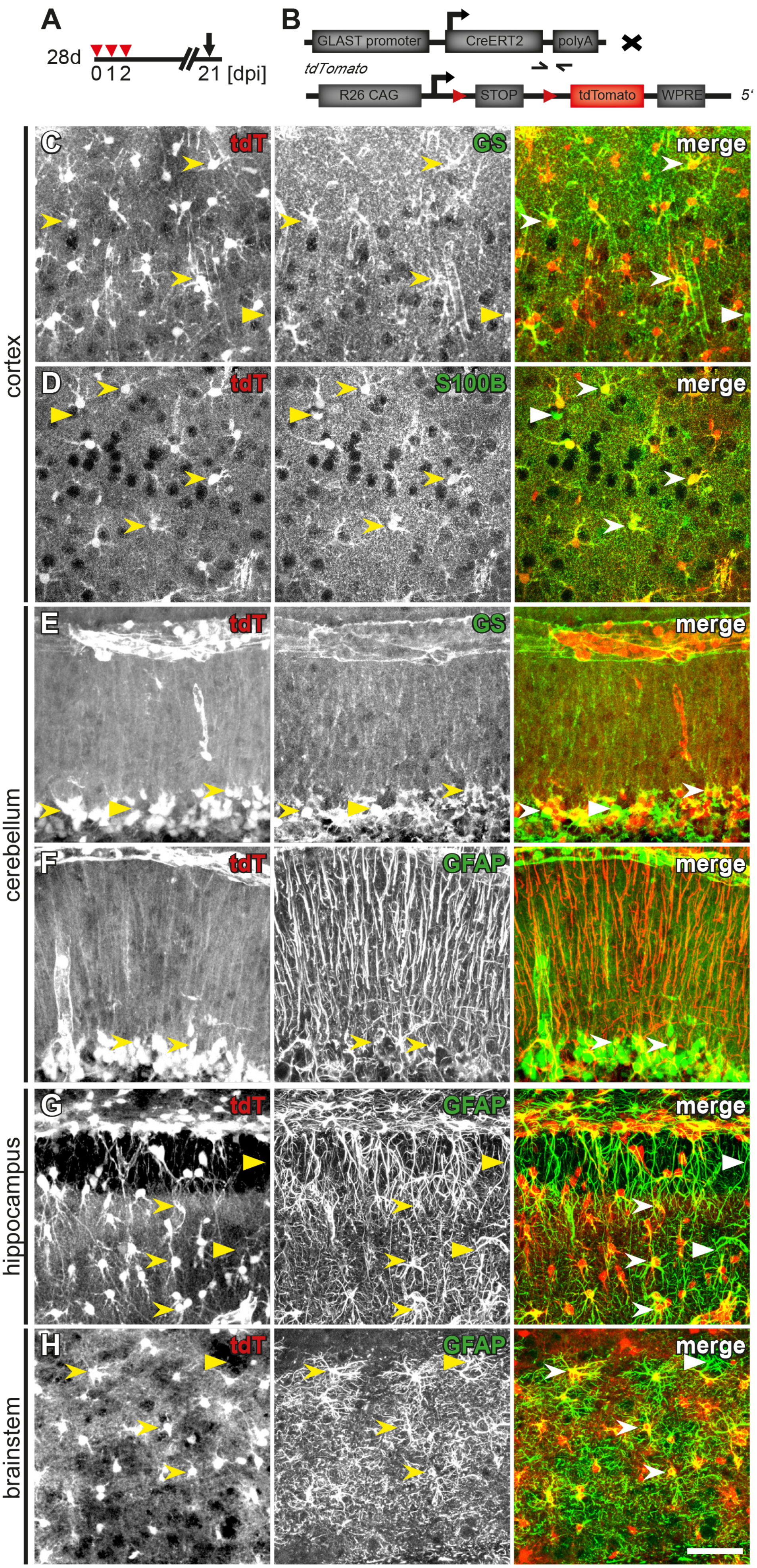

A
28d
0 1 2
21 [dpi]
B
GLAST promoter
CreERT2
polyA
tdTomato
R26 CAG
STOP
tdTomato
WPRE
5‘
cortex
cerebellum
hippocampus
brainstem
C
D
E
F
G
H
tdT
GS
S100B
GFAP
merge

Supplementary information Figure 5: Astrocyte specific recombination of GLAST-CreERT2 x R26-td-Tomato mice in different brain regions. (A) Mice were injected at the age of four weeks with tamoxifen and analyzed 21 d later. (B) Scheme of crossed mouse lines. (C, D) Reporter expression in glutamine synthetase (GS) and S100B-positive astrocytes of the mouse cortex. (E, F) Expression of tdT in GS and glial fibrillary acidic protein (GFAP)-positive Bergmann glia of the cerebellum. (G, H) GFAP-positive astrocytes with tdT expression in hippocampus and brainstem. Co-localization of recombined cells and specific astrocytic markers is indicated by yellow arrowheads, non-recombined astrocytes by yellow triangles. The scalebar in (H) indicates 50 µm and corresponds to all images.

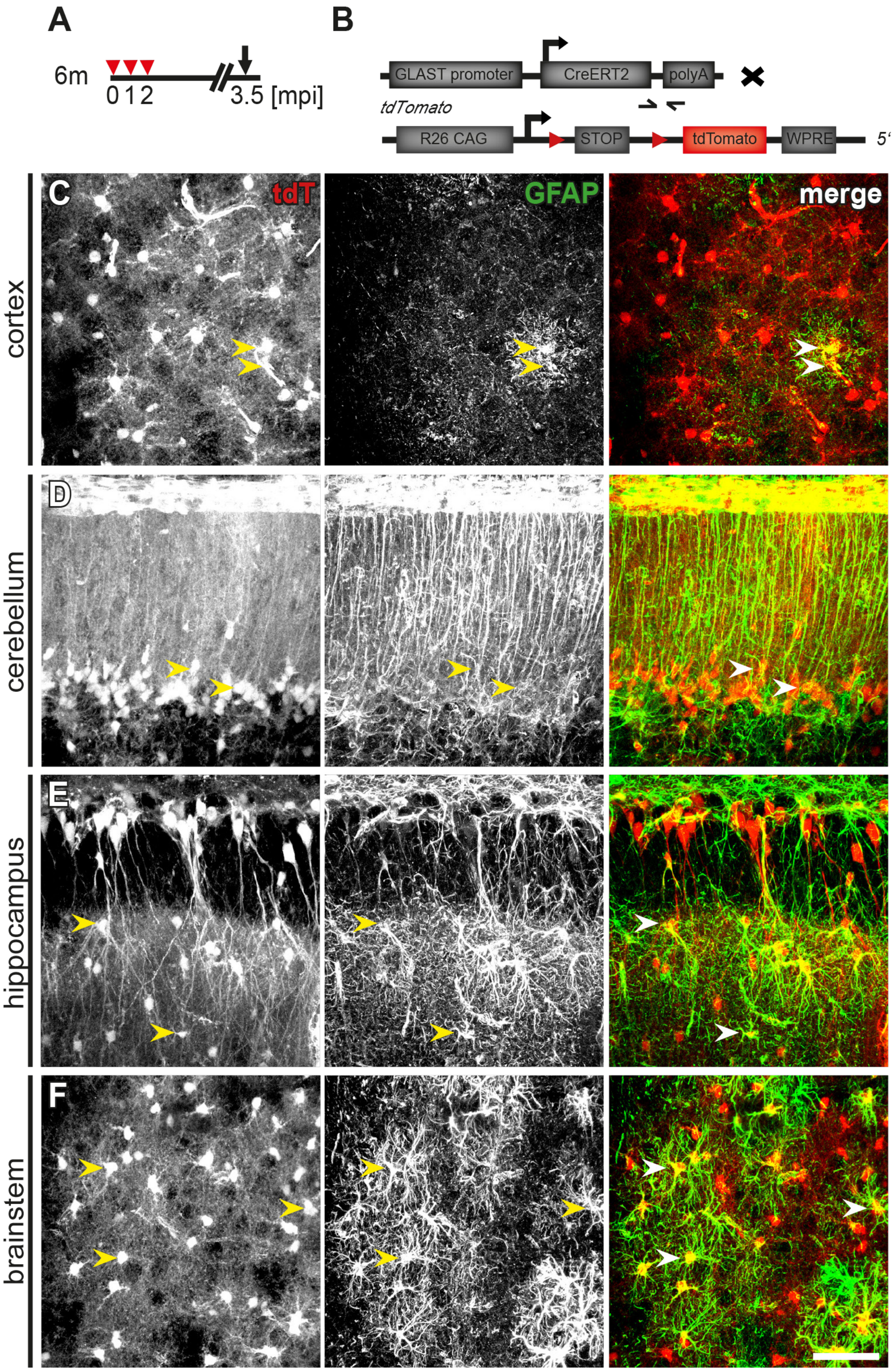

Supplementary information Figure 6: Induction of reporter expression in aged mice revealed high levels of recombination in astrocytes. (A) Mice were injected with tamoxifen at the age of 6 months and analyzed 3.5 months later. (B) Scheme of crossed mouse lines. (C) High tdT expression in cortical astrocytes. GFAP expression was low as expected for this brain region and age of the mouse. (D, E, F) High recombination in cerebellar Bergman glia and astrocytes of hippocampus and brainstem (GFAP positive). Colocalization of recombined cells and GFAP is indicated by yellow arrowheads. The scalebar in (F) indicates 50 µm and corresponds to all images.

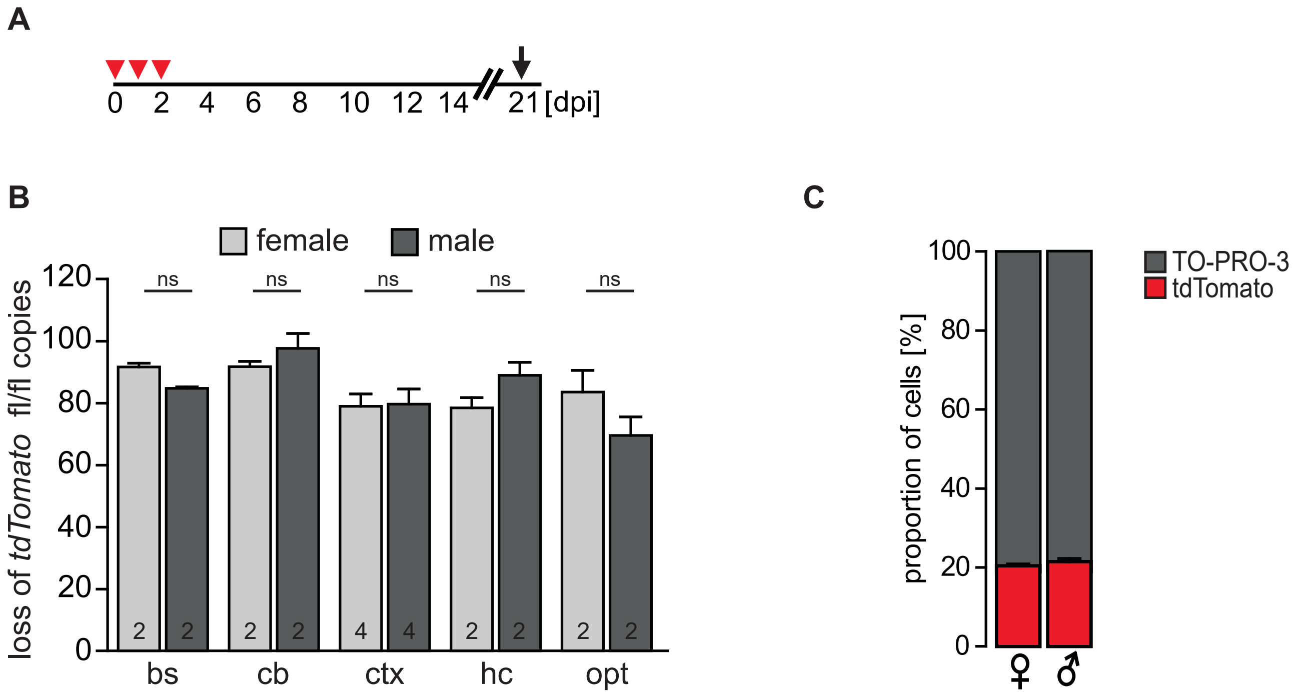

Supplementary information Figure 7: No gender differences in GLAST-driven recombination efficiencies. (A) For the comparison of gender dependent recombination, TAM was injected for three consecutive days (indicated by red triangles) and different brain regions were analyzed at 21 dpi (black arrow). (B) Quantification of floxed non-recombined alleles in bs, cb, ctx, hc and opt by qRT-PCR in female (light grey) and male (dark grey) mice showed no significant differences in all analyzed brain regions. In conclusion, qRT-PCR results of male and female mice were combined in this work. (C) Quantification of TO-PRO-3+ and tdTomato+ cells in cortices of female and male GLAST-CreERT2/+ x stopfl/fl tdTomato vibratom slices resulted in 21 ± 6 % and 20 ± 4 % tdTomato positive cells. The error bars correlate to the SEM of the biological replicates (n = as indicated in the bars, unpaired t-test).

Original dataset for Suppl.Fig1A right panel

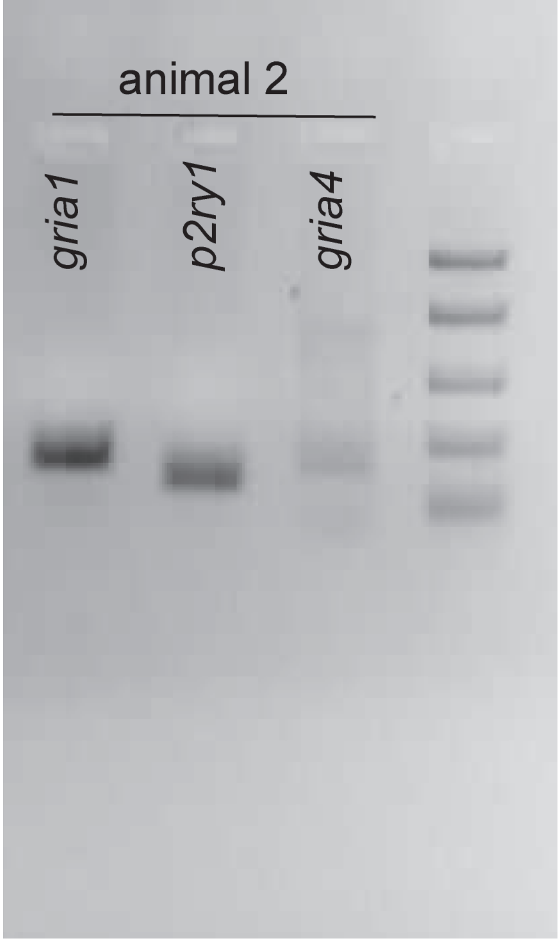

Tables (Supplementary information)

Supplementary information Table 1: Concentrations of TAM and its metabolites in brain and serum detected by LC-HR-MS/MS.

	brain				serum			
**analysis**	**TAM [ng/g]**	**4-OHT-TAM [ng/g]**	**NDM-TAM [ng/g]**	**END [ng/g]**	**TAM [ng/ml]**	**4-OHT-TAM [ng/ml]**	**NDM-TAM [ng/ml]**	**END [ng/ml]**
**single injection**								
1 hpi	3067.76	520.01	67.48	0.90	8744.90	65.66	63.38	1.82
4 hpi	13771.25	2055.34	1269.06	36.12	2949.65	120.06	85.08	16.15
8 hpi	27950.56	4373.59	5555.54	156.19	5479.48	426.71	334.40	89.82
24 hpi	9106.04	2513.90	8690.12	504.79	557.63	304.17	381.41	253.13
2 dpi	1875.14	340.05	3901.47	436.85	183.04	28.53	211.26	131.56
5 dpi	9.17	1.28	1.69	3.63	3.75	0.08		
7 dpi	77.57	12.37	5.21	0	2.44	0.29	0.41	0
14 dpi	0.71	0.87			0.44	0		
21 dpi	3.04	0.86	3.18	0	1.01	0	0.75	0
**3 injections**								
3 dpi	28853.49	5278.55	21889.77	1925.07	2419.20	242.97	763.76	437.54
5 dpi	17375.64	2457.76	15249.50	1867.61	1068.96	161.36	530.05	382.72
7 dpi	317.43	58.35	999.57	142.47	6.05	0.63	7.45	14.13
21 dpi	5.60	1.72	6.63	0	1.65	0.27	1.94	0

Intraperitoneally injected TAM is metabolized in the liver. TAM and its metabolites 4-OH-TAM, NDM-TAM and END can subsequently be found in brain and serum. The table displays the experimental data of all four compounds in ng/g brain as well as ng/ml serum for the given time points. The table gives the values displayed in Figure 2 (brain) and Suppl. information Figure 2 (serum).

Supplementary information Table 2: DNA recombination efficiencies in cortex and cerebellum at different injection protocols of TAM.

	TAM injection	injection intervall	analysis	*p2ry1* [%]	*gria1* [%]
**ctx**	1x	-	8 hpi	14±3	3±1
	1x	-	21 dpi	54±2	20±7
	2x	24 h	21 dpi	50±15	20±11
	3x	24 h	21 dpi	93±1	80±12
	3x	48 h	21 dpi	68±2	39±15
	3x	24 h	204 dpi	79±4	67±6
	5x	24 h	21 dpi	100±5	100±8
**cb**	1x	-	8 hpi	36±1	33±2
	1x	-	21 dpi	55±9	76±2
	2x	24 h	21 dpi	48±1	69±15
	3x	24 h	21 dpi	77±9	87±14
	3x	48 h	21 dpi	74±13	82±4
	3x	24 h	204 dpi	74±1	89±5
	5x	24 h	21 dpi	100±3	101±15

Different protocols of TAM injection give varying recombination efficiencies after 1-5 injections (single injections per day) compared to three injections with a pause of 48 h in between injections. Recombination efficiencies are displayed as relative percentages setting 5x TAM on five consecutive days as 100 %. The table gives the same values as displayed in Figure 3. The errors correlate to the SEM of the biological replicates (n = 2-4, *p<0.05, **p< 0.01, ***p<0.001, ****p<0.0001, unpaired t-test).
